# Spatio-temporal distribution of tuberculosis and the effects of environmental factors in China

**DOI:** 10.1186/s12879-022-07539-4

**Published:** 2022-06-22

**Authors:** Hao Li, Miao Ge, Mingxin Zhang

**Affiliations:** 1 Institute of Healthy Geography, School of Geography and Tourism, Shaanxi Normal University, Xi’an, 710119 China; 2 College of Resources and Environmental Science, Ningxia University, Yinchuan, 750021 China

**Keywords:** Tuberculosis, Spatio-temporal distribution, Geodetector, GWR, Environmental factors

## Abstract

**Background:**

Although the World Health Organization reports that the incidence of tuberculosis in China is decreasing every year, the burden of tuberculosis in China is still very heavy. Understanding the spatial and temporal distribution pattern of tuberculosis in China and its influencing environmental factors will provide effective reference for the prevention and treatment of tuberculosis.

**Methods:**

Data of TB incidence from 2010 to 2017 were collected. Time series and global spatial autocorrelation were used to analyze the temporal and spatial distribution pattern of tuberculosis incidence in China, Geodetector and Geographically Weighted Regression model were used to analyze the environmental factors affecting the TB incidence.

**Results:**

In addition to 2007 and 2008, the TB incidence decreased in general. TB has a strong spatial aggregation. Cities in Northwest China have been showing a trend of high-value aggregation. In recent years, the center of gravity of high-value aggregation area in South China has moved further south. Temperature, humidity, precipitation, PM_10_, PM_2.5_, O_3_, NO_2_ and SO_2_ have impacts on TB incidence, and in different regions, the environmental factors show regional differences.

**Conclusions:**

Residents should pay more attention to the risk of developing TB caused by climate change and air pollutant exposure. Increased efforts should be placed on areas with high-value clustering in future public resource configurations.

## Background

Tuberculosis (TB) is a chronic infectious disease caused by *Mycobacterium tuberculosis*. The disease mainly occurs in the lungs. *The Global Tuberculosis Report 2017* issued by the World Health Organization (WHO) shows that TB is the ninth leading cause of death in the world. Although the statistics show that the global incidence of TB has dropped by 2% every year, the burden of TB is still heavy. According to *China Health Statistics Yearbook 2018*, China is one of the 30 countries with high burden of TB, and the number of TB cases ranks third in the world. The incidence of TB in China declined from 96.31/100,000 in 2005 to 60.53/100,000 in 2017, but the number of TB cases still reached nearly 900,000. WHO has set a global goal of ending TB by 2035. To achieve this goal, the situation of prevention and control in the future is still grim for China.

A large number of studies have shown that the temporal and spatial distribution of TB has complex dynamic characteristics, and the spatial and temporal distribution patterns of TB have different characteristics in different scales. A review of the spatial analysis of TB epidemiology found that methods such as spatial scan statistics and Moran’s *I* spatial autocorrelation analysis were widely used in spatial distribution studies [1]. For example, Rao analyzed the spatial pattern of TB in Qinghai Province of China by using Moran’s *I* spatial autocorrelation analysis and spatial scan statistical method [2, 3]. It was found that the distribution of TB was not random, and had obvious cluster. The same results also occurred in the study of the spatial distribution of TB in Iran and Zhejiang, China, etc. [4–6]. TB also has obvious characteristics on time scale. Sadeq did a long time series study on TB and found that the incidence of TB in Morocco declined from 2005 to 2014 [7]. Although the incidence of TB had been decreasing, it also had seasonal characteristics in the same year. A study of TB in Lahore, Pakistan [8], found that TB has distinct seasonal characteristics. It concluded that the incidence of TB is high in summer and low in winter. A more detailed study of China also showed this feature [6]. The study showed that the highest incidence of TB was in April in 2009, June and July in 2010 and 2011, and the lowest in winter. The seasonal incidence of TB, may be associated with environmental factors. In You’s research [9] on TB in Beijing and Hong Kong, the descriptive analysis and Poisson regression analysis showed that the increase in the number of TB cases notified in the current month was significantly related to the increased monthly PM_2.5_ concentrations.

Climate changes in recent years have led to extreme temperatures, air pollution and other disasters. It was found that the incidence of TB was significantly correlated with regional geographic environmental factors such as temperature, wind speed and air pollution [10]. Keerqinfu [11] established a time series model of TB and meteorological factors in Beijing. A correlation was found between the number of TB cases and meteorological factors and the number of TB cases in the future was predicted by combining seasonal meteorological factors with sarimax model. Fernandes [12] had studied the relationship between climatic factors and air quality with TB in the Brazilian Federal District. The results showed that the meteorological factors such as temperature, humidity, precipitation and the concentration changes of air pollutants such as CO, O_3_, SO_2_ and NO_2_ were the risk factors of TB. Zhu explored the relationship between the incidence of TB and air pollutants in Chengdu, China and concluded that PM_10_, NO_2_ and SO_2_ were positively correlated with the incidence of TB, and there was a lagging effect [13]. A recent study on the relationship between pulmonary TB and SO_2_ in Ningbo found that short-term exposure to low SO_2_ levels may reduce the risk of TB [14].

Even though many regional studies have explored the statistical correlation between environmental factors and the of local TB incidence, and deduced their regional internal correlation, their results are not consistent due to the differences in geographical regions and the limitations of research contents and data base scales. The research on large scale and wide population is relatively weak. The impact of the macro environmental factors on the TB incidence is not clear, and is not conducive to the formulation and implementation of public health policies. Therefore, this study used time series and spatial autocorrelation methods to study the temporal and spatial distribution pattern of TB incidence in China. GeoDetector method was used to explore the correlation between environmental factors and TB incidence in spatial distribution. Geographically weighted regression model (GWR) was used to measure the influence and intensity of environmental factors on TB incidence. This study hopes to provide evidence for developing more rational public health policies and preventing and reducing the TB incidence.

## Methods

### Data sources

The 2005–2017 TB incidence in China was collected from The Data Center of China Public Health Science of Chinese Center for Disease Control and Prevention (CDC). The database used in this study mainly includes the TB incidence in municipal districts of China in 2005–2017 and the TB incidence in different regions and months in China during 2010–2017. The data covers 381 municipal units except Taiwan in China, and there is a lack of data in some areas in a few years.

Geographic information data comes from the database of national basic geographic information center. The temperature, humidity, precipitation, wind speed, air pressure and sunshine hours in the environmental data are derived from the data of 665 meteorological stations of the National Meteorological Science Data Center. PM_10_, PM_2.5_, O_3_, CO, NO_2_ and SO_2_ data come from 1496 national ambient air quality monitoring stations released by China environmental monitoring station. The environmental data is the moving average value in 2017. The data set includes hourly, daily monitoring data for 2017. Some sites have missing or wrong data. This study deletes the date or month of missing data through python (4 h missing every day or 5 days missing every month). Kriging interpolation is performed on all site data to cover the whole study area and partition statistics are carried out.

### Spatial autocorrelation method

Spatial autocorrelation method is used to measure the degree of interdependence between geographic data of a location and other data of the same kind. It used to characterize the spatial concentration and correlation of TB incidence, Moran ‘s *I* was used to measure and characterize its spatial distribution type.

1 $$I=\frac{n}{\sum _{i=1}^{n}\sum _{j=1}^{n}{\omega }_{ij}}\times \frac{\sum _{i=1}^{n}\sum _{j=1}^{n}{\omega }_{ij}({y}_{i}-\stackrel{-}{y})({y}_{j}-\stackrel{-}{y})}{\sum _{i=1}^{n}{({y}_{i}-\stackrel{-}{y})}^{2}}.$$

In the formula: *I* is the global Moran index, *n* is the total number of regions, *i, j* represents a basic unit, ${\omega }_{i,j}$ is the spatial weight, ${\text{y}}_{\text{i}},$ ${y}_{j}$ is the TB incidence in unit *i, j*, $\stackrel{-}{y}$ is the average number of TB incidence. Moran’s *I* index ranges from − 1 to 1, and *I* > 0 indicates positive spatial correlation. The larger the value, the higher the spatial agglomeration. *I* < 0 indicates negative spatial correlation. The smaller the value, the greater the spatial difference. *I* = 0 indicates spatial randomness.

### GeoDetector method

**Fig. 1:**
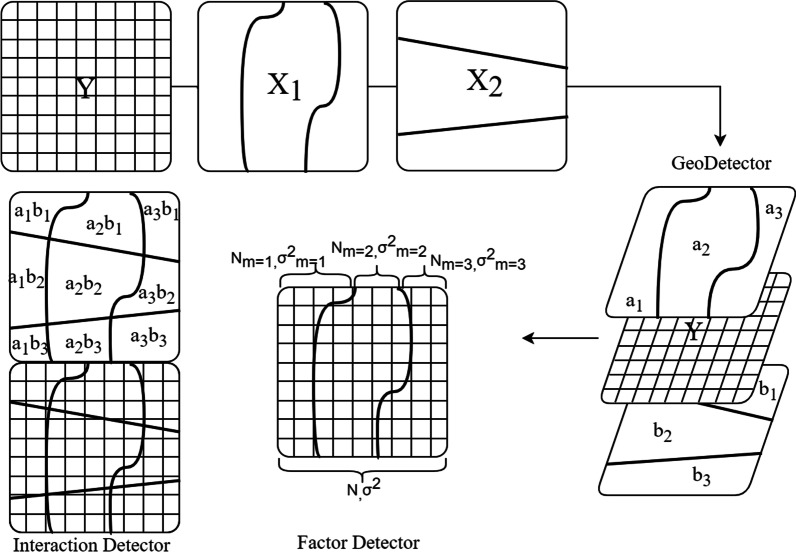
Principle of GeoDetector model

GeoDetector (Fig. 1) is a tool to measure spatial stratification heterogeneity proposed in 2017 [15]. Spatial stratification heterogeneity refers to the difference of an attribute between different types or regions. GeoDetector is based on a geographical perspective: if environmental factors are associated with the TB incidence, their geographical distribution should be similar. The GeoDetector determines or searches for potential environmental factors that affect TB incidence by measuring such similarity. The model requires the input quantity to be category quantity, so it can not be affected by the data distribution characteristics and the multicollinearity between them. In this study, the “GD” package in R4.1.2 software (https://mirror.lzu.edu.cn/CRAN/) was used for GeoDetector analysis [16].The “corrplot” package was used for drawing, and the classification interval of environmental factors is set to 3–8 categories. The spatial discretization method used equal interval, geometric interval, natural discontinuity, quantile, standard deviation, etc. The results were compared to find the optimal model. Table 1 shows the optimal classification results of environmental factors.

**Table 1:** Environmental factors and the classifications

Environment factors	Index	Classification
Meteorological factors	Temperature	7
	Wind Speed	8
	Humidity	7
	Pressure	7
	Precipitation	6
	Sunshine hours	6
Air pollutants	PM_2.5_	8
	O_3_	8
	CO	8
	PM_10_	7
	NO_2_	5
	SO_2_	7

The factor detection is used to detect spatial heterogeneity of TB incidence and to explore its geographical similarity with environmental factors. It is measured by *q* value.

2 $$q=1-\frac{1}{N{\sigma }^{2}}\sum _{m=1}^{L}{N}_{m}{\sigma }_{m}^{2},$$

where *q* is a similarity between explanatory variables and geographic distribution of TB incidence; *L* is the total number of categories of variables; *m* is the category of explanatory variable and dependent variable; *N* is the number of units in all areas;${\sigma }^{2}$ is the variance of the dependent variable; *N*_*m*_ represents a category of explanatory variables, ${\sigma }_{m}^{2}$ is the variance of this category. The range of *q* value is [0,1]. The larger the *q* value, the greater the geographical distribution similarity between the explanatory variable and the dependent variable, indicating the greater the potential impact on the dependent variable.

The occurrence of disease is a comprehensive process. Different influencing factors may have synergistic or antagonistic effects on the impact of disease. The interaction between the two factors will weaken or enhance the impact of a single factor on the disease. Interaction detection calculates the *q* value *q*(*x, y*) when the spatial distribution of the two influencing factors is superimposed, and compares *q*(*x*), *q*(*y*) and *q*(*x, y*) to determine the type of interaction (Table 2).

**Table 2:** The type of factor interaction expression

Formula	Interaction
*q*(*x*, *y*) < Min(*q*(*x*),*q*(*y*))	Weaken, nonlinear
Min(*q*(*x*), *q*(*y)*) < *q*(*x*, *y*) < Max(*q*(*x*),*q*(*y*))	Weaken, unilateral
*q*(*x*, *y*) > Max(*q*(*x*),*q*(*y*))	Enhance, bi-linear
*q*(*x*, *y*) = *q*(*x*) + *q*(*y*)	Independent
*q*(*x*, *y*) > *q*(*x*) + *q*(*y*)	Enhance, nonlinear

### Geographically weighted regression (GWR) model

GWR model is a linear regression model. Compared with general linear regression models, the results increase local regression coefficients based on different geospatial divisions. GWR model can get the difference of local impact of environmental factors on TB incidence in different geographic locations, which can make up for the disadvantage of GeoDetector model in not measuring the positive or negative impact of environmental factors on geospatial distribution, and determine the impact intensity of local environmental factors. This study uses the GWR tool in ArcGIS10.2 software (https://www.esri.com/en-us/home) to model relationships between environmental factors and TB incidence.

## Results

### Time pattern of TB incidence

**Fig. 2:**
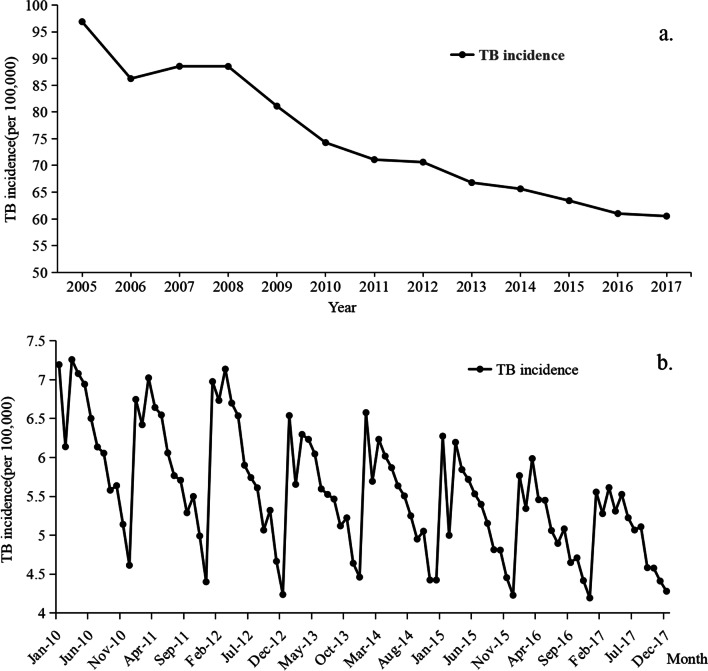
Time series of TB incidence in China(a. Annual changes in TB incidence in China from 2005 to 2017, b. Monthly variation of TB incidence in China from 2010 to 2017)

Figure 2a shows the temporal variation of tuberculosis in China from 2005 to 2017. In addition to 2007 and 2008, the TB incidence decreased in general. The TB incidence dropped from 96.878/100,000 in 2005 to 60.528/100,000 in 2017, a decrease of 37.5%. This shows that in recent years, the national policy and governance of tuberculosis is very effective. According to the characteristics of TB incidence in recent years (2010–2017, Fig. 2b), it shows that the TB incidence is obviously seasonal. It was the highest in spring, followed by winter and the least in autumn. According to the monthly incidence curve, The TB incidence in a year has a very obvious time rule. It was the highest in January, and was the lowest in December, and the incidence decreased significantly in February, which may be related to the duration of statistics. The TB incidence decreased gradually after a short increase in March.

### Spatial patterns of TB incidence

#### Geographical distribution of TB incidence

**Fig. 3:**
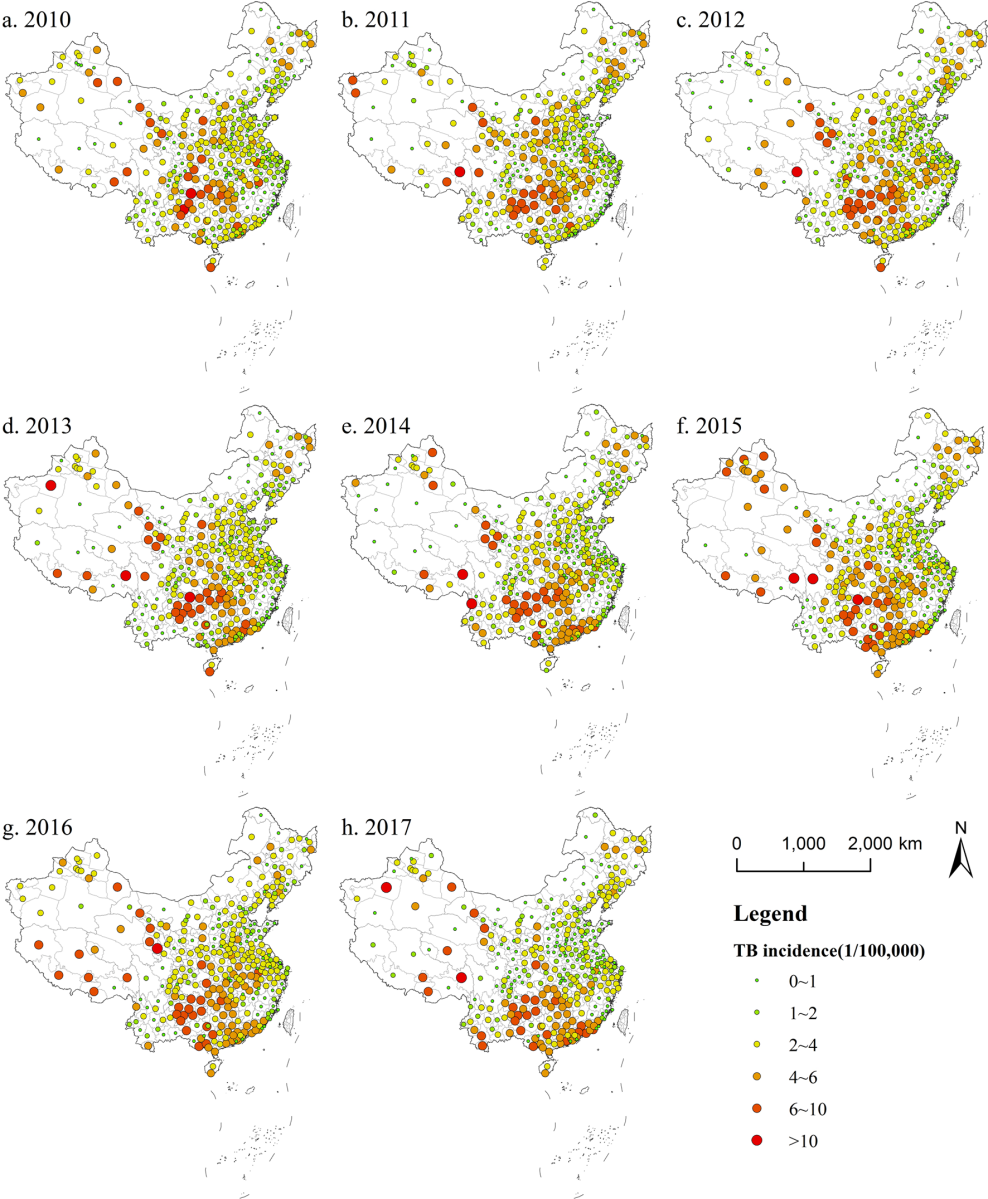
2010–2017 spatial distribution of TB incidence in China (Created by ArcGIS 10.2 software https://www.esri.com/en-us/home)

Figure 3 shows the geographical distribution and change of the TB incidence in China during 2010–2017. It can be seen clearly that although the TB incidence of China was decreasing every year, it had different weight in different regions in China. The TB incidence in most cities in eastern and central China decreased in 2010–2017. The TB incidence in Western China has fluctuated greatly, and there is a trend of growth in some areas. This may also be related to statistics. Statistical means are constantly improving, and the population of statistics is more complete, which may also cause an abnormal increase in TB incidence. Overall, TB incidence in eastern coastal and northern regions is relatively low. The TB incidence in some areas of northwest and south is higher, and the burden of disease is heavier. The TB incidence increases from east to west, from north to south.

The high TB incidence is in Western China. Through statistics of the top 5 places of the TB incidence in 2010–2017 (Table 3), it can be found that among the 40 cities, except Danzhou, the remaining 39 cities are located in Western China, which shows that the western region is a key area that needs to strengthen the prevention and control of TB in the future.

**Table 3:** The top 5 places of the TB incidence in 2010–2017

Year	City	TB incidence (1/100,000)	Year	City	TB incidence (1/100,000)
2010	Zunyi	12.6052	2014	Golog	22.8361
	Anshun	10.1016		Yushu	21.7543
	Chamdo	9.3887		Nujiang	11.1318
	Sanya	8.2005		Chamdo	10.1936
	Turpan	8.1043		Zunyi	9.8088
2011	Chamdo	10.4937	2015	Golog	31.736
	Zunyi	9.8562		Kashi	27.8275
	Anshun	8.9219		Yushu	26.9258
	Ganzi	8.8182		Haidong	18.2254
	Qiannan	8.6769		Kizilsu	17.582
2012	Yushu	25.5355	2016	Golog	41.2223
	Golog	20.9388		Yushu	29.4183
	Chamdo	12.067		Haidong	19.1456
	Zunyi	9.3517		Turpan	18.7274
	Anshun	9.1686		Huangnan	11.5778
2013	Kashi	22.1511	2017	Haidong	20.9997
	Golog	20.2144		Danzhou	20.4288
	Kizilsu	14.9391		Turpan	19.6916
	Aksu	11.775		Kizilsu	14.3752
	Zunyi	10.2794		Golog	13.0049

#### Spatial aggregation

In order to quantitatively evaluate the spatial distribution and aggregation of TB in China, The global spatial autocorrelation analysis and Getis-Ord Gi* statistics analysis were used to calculate the global Moran’s I index and the incidence rate hotspots. The TB incidence in China shows a clear spatial aggregation. The Moran’s I index was used to quantify the spatial clustering of diseases. The results (Table 4) show that the Moran’s I is in the range of 0.107–0.225 in 2010–2017, and the z scores are all greater than 2.58 (p < 0.01), which means that the spatial distribution of TB incidence tends to be clustered. The Z-score represents a multiple of the standard deviation.

**Table 4:** Global spatial autocorrelation analyses for TB incidence of China from 2010 to 2017

Year	Moran’s *I*	Z Score	P value	Pattern
2010	0.140	9.995	< 0.01	Clustered
2011	0.225	15.899	< 0.01	Clustered
2012	0.131	9.634	< 0.01	Clustered
2013	0.129	9.414	< 0.01	Clustered
2014	0.177	12.867	< 0.01	Clustered
2015	0.161	11.802	< 0.01	Clustered
2016	0.107	8.319	< 0.01	Clustered
2017	0.203	14.634	< 0.01	Clustered

**Fig. 4:**
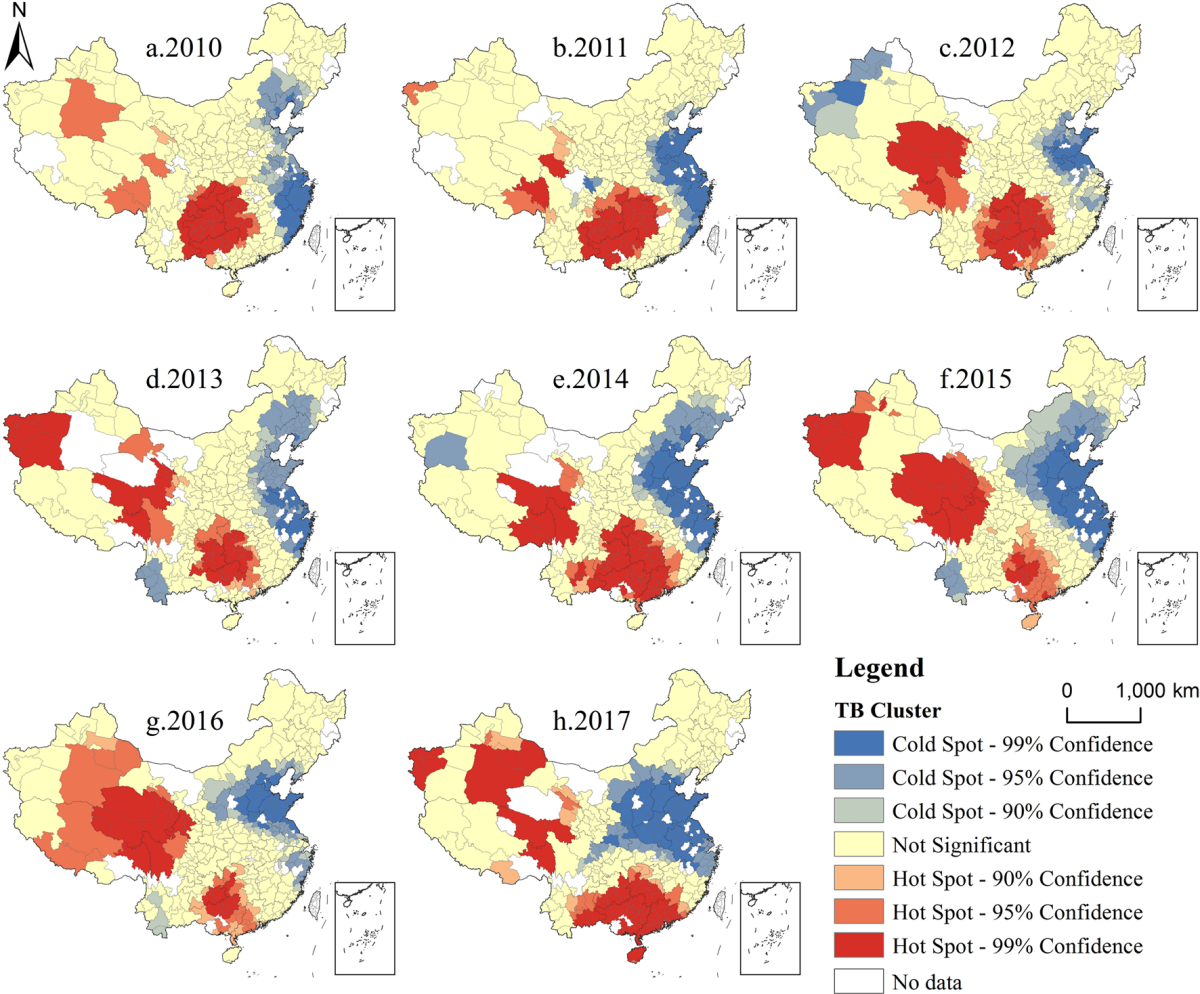
The spatial clusters of the TB incidence in China (Created by ArcGIS 10.2 software https://www.esri.com/en-us/home)

Based on the global spatial autocorrelation analysis, the Getis-Ord Gi* statistics analysis was used to identify the hotspots and coldspots of the TB incidence. Hot spots represent that a location element has a high value and is surrounded by other elements with the same high value. The local sum of a feature and its adjacent features will be compared with the sum of all features; When the local sum is very different from the expected local sum, the Z score will be calculated. If the Z score is positive, it represents the hot spot area, and the cold spot area is the opposite. Figure 4 shows the cold and hot spots of TB incidence in China in 2010–2017, respectively, which was in 90%, 95% and 99% confidence levels. There are two hot spots of TB incidence in China, located in the northwest and south. Cities in Northwest China have been showing a trend of high-value aggregation. In recent years, the center of gravity of high-value aggregation area in South China has moved further south. The cold spot area is located in the eastern coastal area, and some cities in Southwest China once showed the characteristics of cold spots. From Fig. 4, it is obvious that the TB incidence is increasing gradually, and the coastal area has been extended inland from the east coast in recent years.

### Correlation of environmental factors and TB incidence

#### Results of GeoDetector model

**Fig. 5:**
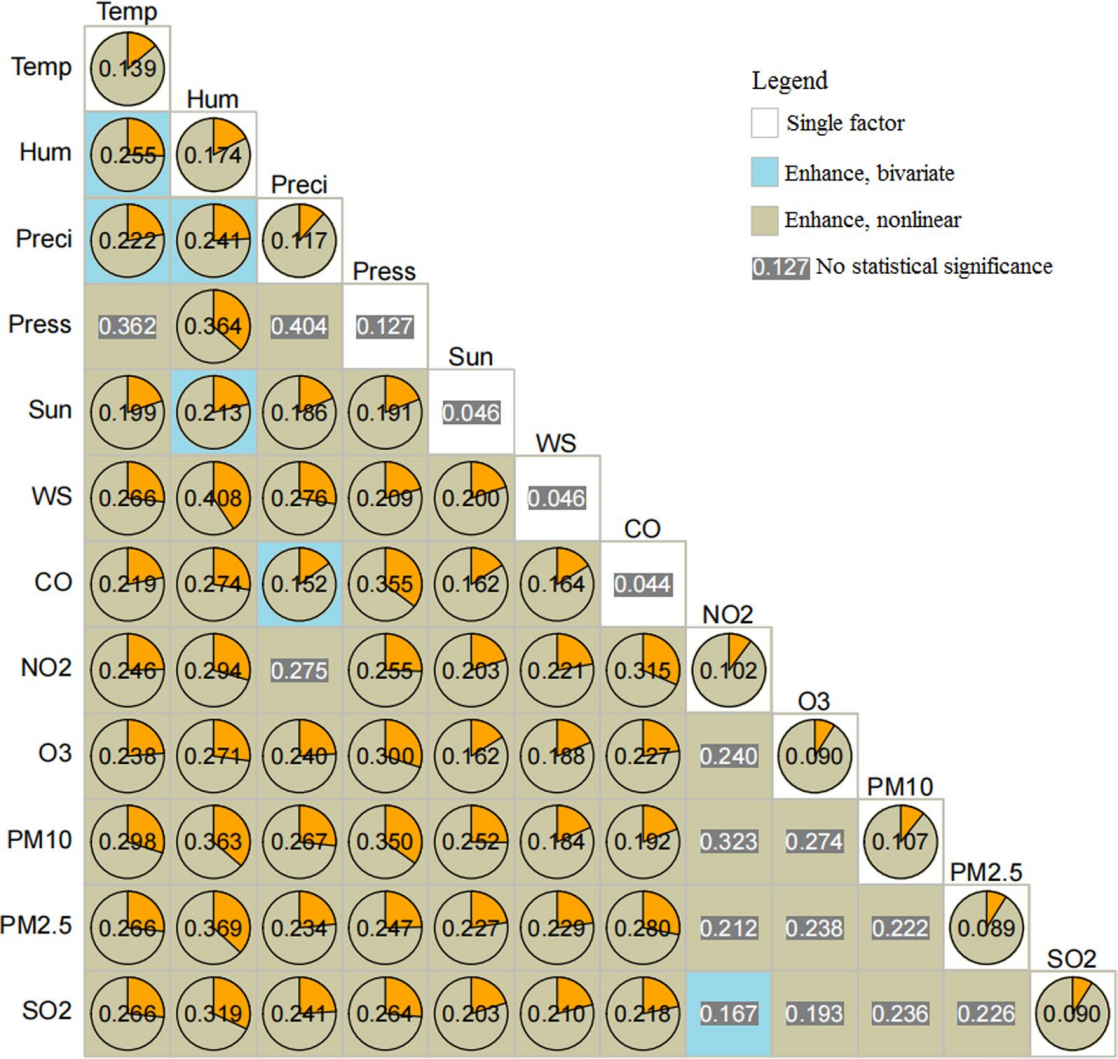
GeoDetector results for association of environmental factors with TB incidence (Temp: temperature; Hum: humidity; Preci: precipitation; Sun: sunshine hours; WS: wind speed. Created by R4.1.2 software https://mirror.lzu.edu.cn/CRAN/)

Based on GeoDetector model, the 2017 TB incidence and environmental factors were selected as samples to explore the similarity between the spatial distribution of them. Figure 5 shows the results of GeoDetector model. Among the selected environmental factors, air pressure, sunshine hours, wind speed and CO did not pass the significance test of single factor detection. This indicates that there is no obvious correlation between these environmental factors and TB incidence in the perspective of geographical distribution. Among the 8 environmental factors examined, the q value of humidity was the largest (0.174), indicating that the humidity factor had the highest geographical distribution similarity with TB incidence, and it had the highest potential impact. The order of environmental factors is humidity > temperature > precipitation > PM_10_ > NO_2_ > SO_2_ > O_3_ > PM_2.5_. Influence of meteorological factors on TB incidence is stronger than that of air pollutants.

The interaction results between environmental factors are shown in Fig. 5. The interaction between any two factors plays an important role in the potential impact of TB incidence. After excluding the environmental factors that fail to pass the significance test by student’t test, it is concluded that the influence intensity of the interaction of various environmental factors is stronger than that of single factor, and there is an enhancement effect between environmental factors. Most environmental factors have nonlinear enhancement effects, which indicates that environmental factors have obvious synergistic effects, and the internal relationship between environment and TB may be more complex. The interaction between humidity and wind speed (0.408) is the highest similarity with the geographical distribution of TB incidence. The interaction between humidity and air pollutants has a stronger influence on TB incidence than most other factors. This indicates that areas with obvious humidity and air pollution characteristics need more attention.

#### Results of GWR model

**Fig. 6:**
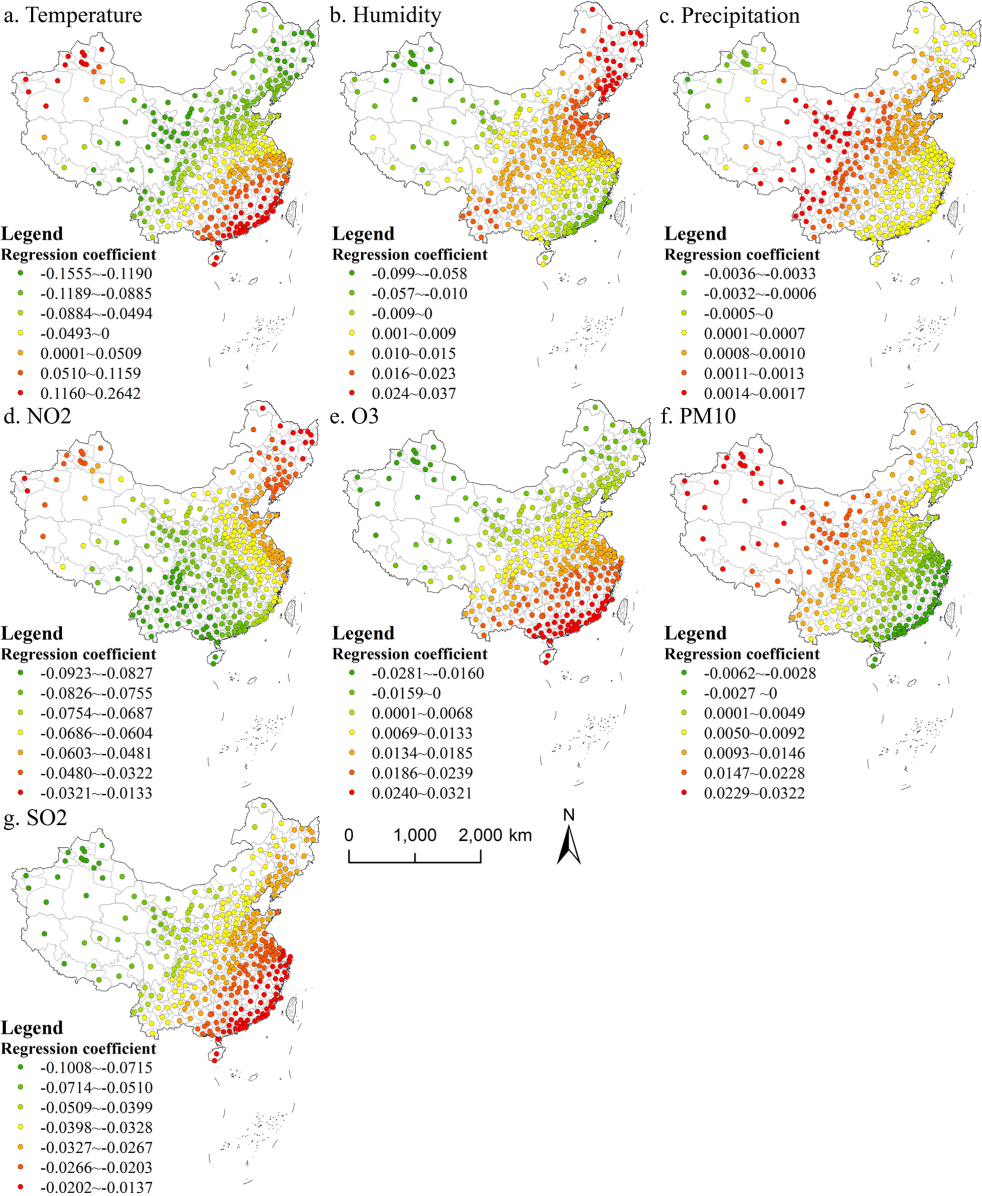
GWR results on the impact of environmental factors on TB incidence in 2017 (Created by ArcGIS 10.2 software https://www.esri.com/en-us/home)

Many studies show that the environmental factors in different regions may have different degrees of influence on TB incidence, or even the opposite trend [17–19]. Therefore, GWR model was used to further measure the positive or negative effects of environmental factors on TB incidence and their intensity under different geographical locations. The ordinary least squares model (OLS) was first used to eliminate the multicollinearity between various environmental impact factors. The results show that there is collinearity between PM_10_ and PM_2.5_ that exceeds the requirements of GWR Model (VIF > 7.5). Therefore, PM_10_, which is more representative, was selected as the main factor in this study. The GWR results (Fig. 6) show that the temperature factor shows a local positive impact in Southeast and Northwest China, and the other regions are negatively correlated. Contrary to temperature, humidity was negatively correlated in Southeast and northwest regions, and the rest areas were positively correlated with TB incidence. Only Northwest China’s precipitation has negative effects on the incidence rate of tuberculosis. The precipitation in other areas is positively correlated and shows a trend of increasing from east to west. SO_2_ and NO_2_ have negative effects on TB incidence in the whole country. The impact of NO_2_ on TB incidence increased from Southwest China to northeast and northwest. The influence of SO_2_ showed a decreasing trend from Southeast China to northwest. Among other air pollutants, O_3_ has a difference in TB incidence, and has a positive effect in Southern China and negative in North China. On the contrary, the impact of PM_10_ is negatively correlated in the eastern coastal area, positively correlated in the inland area and gradually increasing to the northwest.

## Discussion

Since WHO put forward the goal of terminating tuberculosis, China has also responded positively, and implemented measures such as strengthening TB propaganda and Issuing the action plan to curb TB (2019–2022), and achieved remarkable results. The study on the spatial distribution pattern of TB incidence and the environmental impact will provide important reference for effective prevention and treatment of TB and national policy formulation. With the rapid development of economy, China’s TB prevention and control has also achieved great results. However, driven by industrialization and urbanization, the uneven socio-economic distribution and the aggravation of air pollution in some areas have emerged one after another, which will have a great impact on the prevention and control of TB.

The TB incidence of China has been decreasing since 2005, which shows that China’s policy implementation over the years has been very fruitful. The TB incidence has obvious seasonal characteristics in time, which is the same as other regional studies [20, 21]. On the monthly scale, due to the difference of regional scale, TB incidence in China was the highest in January, and decreased in February. It rose in March, then continued to decline, and the TB incidence in December was the lowest. Other regional studies have found that TB incidence has similar time patterns in different regions of China [13, 22]. Overall, measures should be taken in spring and winter to prevent the high TB incidence. Most studies focus more on the time series of regional TB incidence, while few studies on the spatial and temporal distribution of TB incidence nationwide. This study found that China’s TB incidence has two distinct high value aggregation areas, which is similar to the results of an earlier national study [23]. The same studies also showed that TB cases are clustered [24, 25]. More importantly, this study found that in recent years, the center of gravity of the aggregation area in Southern China has gradually moved further south.

Relevant research shows that TB is a disease related to poverty [26]. Therefore, many studies pay more attention to the impact of regional economic factors on local TB incidence, thus ignoring the impact of environmental factors. For a long time, TB incidence has shown seasonal changes. Scholars speculate that this may be related to environmental factors [9]. This study combines the GeoDetector with GWR to explore potential environmental factors affecting TB incidence from spatial distribution perspective and quantify their relationship. The study found that temperature, humidity and precipitation were related to TB incidence. According to the GWR results, the appropriate temperature was the protective factor for TB. Studies have shown that temperature can inhibit the growth of Mycobacterium tuberculosis, but too high or too low temperature can increase the risk of TB [27]. Humidity and precipitation will increase the risk of TB, but there is a negative correlation between humidity and precipitation in the northwest polar arid region of China. A regional study on Northwest China also found a negative correlation [22]. The influence mechanism of meteorological factors on the pathogenesis of TB is not completely clear. Pinto found that the Mycobacterium tuberculosis group will stop growing at 37℃, so when the temperature is higher, it will reduce the risk of TB, and sunshine in summer can increase the synthesis of vitamin D and increase human immunity [27]. Baughman found that humidity will affect people’s circulatory system, thus increasing the sensitivity of the human body to infectious diseases [28]. Moist air is conducive to the survival and reproduction of Mycobacterium tuberculosis, and increases the risk of TB [29, 30]. Through experiments, scholars can only partially explain the biological processes affected by meteorological factors, and its internal occurrence and development mechanism still needs to be further studied.

The impact of air pollutant exposure on human health has been concerned by many scholars [31–33]. In recent years, with the transformation of the characteristics of air pollution from soot pollution to compound pollution, the impact of air pollutants on human body has gradually appeared. This study found that in addition to CO, the other air pollutants (PM_10_, PM_2.5_, O_3_, NO_2_ and SO_2_) were all associated with TB incidence. Studies have shown that exposure to high concentrations of inhalable particles and O_3_ will cause airway hyperresponsiveness and DNA damage, which will damage the human respiratory system, making it easier for infectious bacteria to enter the human body and lead to diseases [34, 35]. However, no association were found in some low pollution areas [14]. This is consistent with the local results of this study. In particular, the study found that NO_2_ and SO_2_ were negatively correlated with TB incidence, which is inconsistent with the general findings [36, 37]. It is generally believed that air pollutants will damage the human respiratory system and cause inflammatory reaction and oxidative stress [13]. However, some research evidences are the same as the conclusions of this study. The relevant research results show that low concentrations of SO_2_ and NO_2_ exposure may lead to oxidative damage of lipids and proteins, activate human cells against Mycobacterium tuberculosis, and then reduce the risk of TB [14, 38]. There is an obvious interaction between air pollutants in affecting TB incidence. This study found that the interaction model could better explain the association between air pollutants and TB incidence. The study also showed that the joint action of air pollutants and meteorological factors would directly or indirectly affect the risk of TB [39]. Although the study of TB incidence and air pollutants has attempted to partially explain its impact mechanism, the research results need to be further integrated and deepened due to the differences in data accuracy and research methods.

This study has certain significance for national policy-making, optimizing the allocation of public health resources and realizing the goal of ending tuberculosis. Meanwhile, this study also has some disadvantages. First, after pooling all TB cases within one aggregate region (administrative district boundaries), the scale effect may make the error larger and some correlations masked. And because of the scale effect, the influence of environmental factors may have an imprecision with the real situation. Second, we have not been able to capture data on a smaller scale, such as counties or neighborhoods, so we can’t study it in more detail. Third, this study did not consider the effect of economic factors on TB incidence, and the corresponding medical conditions in economically developed regions are more perfect and more timely and efficient treatment for TB, which may also lead to a bias in the final result of the effect of environmental factors on TB incidence.

## Conclusions

This study analysed the spatio-temporal distribution pattern of TB incidence in China and explored the differences in the regional effects of environmental factors on TB incidence. There are regional differences in the effect of environmental factors on TB incidence, residents should pay more attention to the risk of developing TB caused by climate change and air pollutant exposure. There is strong spatial clustering of TB incidence, and it is recommended that increased efforts should be placed on areas with high-value clustering in future public resource configurations.

## Data Availability

The data that support the findings of this study are available from the Data-center of China Public Health Science repository (https://www.phsciencedata.cn/Share/edtShareNew.jsp?id=39208) but restrictions apply to the availability of these data, which were used under license for the current study, and so are not publicly available. Data are however available from the authors upon reasonable request and with permission of the Data-center of China Public Health Science. This data set can also be applied for by registering an individual account and is free of charge. At present, partial datasets generated and analysed during the current study are not publicly available due to the sharing policy but are available from the corresponding author on reasonable request.

## Abbreviations

TB: Tuberculosis
WHO: World Health Organization
CDC: Chinese Center for Disease Control and Prevention
GWR: Geographically weighted regression
OLS: Ordinary least squares model
CO: Carbon monoxide
O_3_: Ozone
NO_2_: Nitrogen dioxide
SO_2_: Sulfur dioxide
PM_10_: Particulate matter with particle size below 10 μm
PM_2.5_: Particulate matter with particle size below 2.5 μm
